# Assessment of health-related quality of life in Sudanese children with nephrotic syndrome: a questionnaire-based study

**DOI:** 10.11604/pamj.2022.43.154.34980

**Published:** 2022-11-24

**Authors:** Nahla Allam, Asmaa Bashar, Riham Eid

**Affiliations:** 1 Noura Center for Pediatric Kidney Diseases and Renal Transplantation, Al Neelain University, Khartoum, Sudan,; 2 Pediatric Nephrology Unit, Mansoura University Children’s Hospital, Faculty of Medicine, Mansoura University, Mansoura, Egypt

**Keywords:** Quality of life, nephrotic, children, Sudan

## Abstract

**Introduction:**

nephrotic syndrome (NS) is a common glomerular disease in children. The long relapsing nature of the disease along with medication-related complications can affect all aspects of the life of the affected children. This study points to estimate the health-related quality of life (HRQOL) in Sudanese children with NS.

**Methods:**

this case-control questionnaire-based descriptive study included 100 children with NS aged 2-18 years and 100 healthy children. HRQOL was assessed using the Arabic copy of the Pediatric Quality of Life Inventory Generic Core Scales (PedsQL™ 4.0 GCS).

**Results:**

most of the patients were males (64%) and 17% were steroid resistant. Mean PedsQL™ 4.0 summary and domains´ scores in NS were significantly lower than controls (p≤ 0.05 for all) except for the social domain (p=0.266) with the lowest patients´ scores being for school functioning (mean ± SD, 74.4 ± 26.4). The QOL scores considerably differed between the various clinical phenotypes of NS with patients in the initial episode (N=25) having considerably lower total, school, and social domains scores compared to other clinical types (p=0.027, 0.017, 0.006 respectively).

**Conclusion:**

this study assesses for the first time the QOL in Sudanese children with NS. Sudanese children with NS had lower life quality scores in comparison to healthy children and need tireless efforts to improve their lives. PedsQL™ 4.0 scale is simple and can be used in everyday clinical practice to evaluate QOL in children with NS.

## Introduction

Nephrotic syndrome (NS) is a common glomerular disorder in children with most cases being steroid responsive with satisfactory long-term consequences [1]. However, in Africa, children have a special pattern of NS with the majority being steroid-resistant with a higher threat of deterioration to renal damage [2]. Commonly most steroid-sensitive NS children experience one or more relapses [3,4].

The extended path of the disease, recurring relapses, hospital stay, and use of drugs have a huge impact on all features of the life of NS children. Hereafter, a formal appraisal of the influence of the disease on emotional, social, physical, and school performance and health related QOL (HRQOL) is imperative to deliver comprehensive patient care [5,6]. Reports on HRQOL of NS children from developing countries are insufficient [7,8]. Bearing in mind the scarceness of information on QOL in these patients in general and Sudanese children particularly, we carried out the current research.

The objective of the current work is to appraise the HRQOL in children with NS in comparison to controls and delineate the HRQOL fields in diverse clinical subtypes of NS using PedsQL™ 4.0 Generic Core Scales (GCS). We predict that this data will provide a precious point of reference for providing awareness for children and their relatives to help relieve the problems they face and recover their life quality.

## Methods

**Study design and settings:** this is a case-control questionnaire-based study. The study was conducted at Soba University Hospital, Khartoum, Sudan between November 2020 and June 2021.

**Participants:** the cases included 100 children with NS aged 2-18 years with normal kidney function defined as estimated glomerular filtration rate (eGFR) above 90 ml/min/1.73 m^2^ [9] and with no edema, or infection, and not hospitalized at the time of the study. Children with disabilities or psychological difficulties diagnosed before the beginning of NS were eliminated. The controls included 100 age and sex matched healthy children.

**Definitions:** nephrotic syndrome was defined as stated per the “International Study for Kidney Diseases in Children” criteria as heavy proteinuria, hypoalbuminemia (serum albumin < 2.5 g/dL), hyperlipidemia (serum cholesterol > 200 mg/dL), and edema [10]. Relapse was defined as nephrotic range proteinuria for more than 3 consecutive days [10]. Active NS, steroid resistant nephrotic syndrome (SRNS), steroid-dependent nephrotic syndrome (SDNS), frequent relapsing nephrotic syndrome (FRNS), and infrequent relapsing NS (IFRNS) are demarcated as per the Kidney Disease Improving Global Outcomes (KDIGO) guidelines [11].

**The PedsQL™ questionnaire:** an “Arabic” form of the PedsQL™ 4.0 Generic Core Scale (GCS) was used. Patients´ data were extracted from the hospital records. The instrument evaluates the QOL in five domains: physical, emotional, social, and school functioning with higher scores revealing better QOL. The acquired results from our patients and controls in all fields of PedsQL™ then paralleled to its published reference data [12].

**Sample size:** at the time of study 120 patients with idiopathic nephrotic syndrome (INS) were on routine follow-up in our hospital over the former year. So, the population size is 120, the significance level of 0.05 the calculated sample size was 93. Fifteen percent of the sample size (13.9) was added to counterbalance for lacking data. The final size was 107 but 7 patients were not incorporated due to unwillingness. The control group was chosen to be equal to the patients´ group.

**Statistical methods:** data were analyzed using SPSS 25.0 and reported as descriptive statistics in terms of frequency tables with percentages and means of standard deviation. Pearson chi-square or Fisher´s exact test was used for assessing variances in categorical data between groups. Student´s t-test and Mann-Whitney test were used for parametric and non-parametric respectively.

**Ethical consideration:** all patients/guardians postulated knowledgeable consent before enrolment in the study. The study protocol was reviewed and approved by the Ministry of Health and Sudan Medical Specialization Board (SMSB) Ethical Committee.

## Results

**Participants:** two hundred children joined the study, 128 males with patients´ age mean of 9.6 ± 4 years. Data of study participants are shown in Table 1. All NS children had normal kidney function at the time of enrollment measured by eGFR which was 97.1 ± 2.7 ml/min/1.73 m^2^.

**Table 1:** clinical data of the studied patients and controls

	Nephrotic patients N=100	Controls N=100	p-value
Age at enrolment in the study (mean ± SD)/years	9.6 ± 4.03	8.9 ± 5.1	0.282
Male/female	64/36	64/36	1
Age at diagnosis (mean ± SD)/years	8.2 ± 3.8		
Duration of illness (months) (mean ± SD)	16.2 ± 12.9		
**Clinical types of NS: N/%**			
Initial diagnosis	25(25%)		
SDNS	17(17%)		
SRNS	13(13%)		
FRNS	22(22%)		
IFRNS	23(23%)		
**Complications: N%**			
Obesity	20(20%)		
Hypertension	12(12%)		
Diabetes	0		
Cushingoid	17(17%)		
**Medications: N/%**			
Steroids only	33(33%)		
Steroids + levamisole	5(5%)		
Steroids + MMF	25(25%)		
Steroids + cyclophosphamide	9(9%)		
Steroids + cyclosporine	28(28%)		

SD: standard deviation; NS: nephrotic syndrome; SDNS: steroid dependent nephrotic syndrome; SRNS: steroid resistant nephrotic syndrome; FRNS: frequent relapsing nephrotic syndrome; IFRNS: infrequent relapsing nephrotic syndrome; MMF: mycophenolate mofetil

**Main data:** the scores for all domains are presented and matched between patients and controls in Table 2 and showed that the scores of all PedsQL™ 4 GCS domains in the patients were significantly lower than the control group apart from the social domain. The social domain comprehensive questions comparison showed the problem with “other children not desiring to be his or her friend” and “not able to do things that other kids can do” to be considerably lower in cases compared to controls. Table 3 shows scores for all PedsQL™ 4-GCS domains for patients and controls in comparison to normative data and showed that normative data are significantly lower than both cases and controls´ scores for all fields. Comparing the clinical phenotypes of NS disclosed that firstly presented children with NS have the lowest scores for the entire score, social and school domains (p < 0.05) compared to other clinical forms, whilst physical and social domains´ scores did not vary between different phenotypes of NS (Table 4).

**Table 2:** PedsQL™ 4.0 GCS scores in cases and controls

Domain	Nephrotic patients		Controls		p-value
	Mean ± SD	Median	Mean ± SD	Median	
Physical	90.8 ± 13.3	93.7	95.6 ± 10.5	100	0.005
Emotional	86.8 ± 13.4	90	91.6 ± 14.7	100	0.017
Social	95.8 ± 10.9	100	97.3 ± 7.9	100	0.266
School	74.4 ± 26.4	85	90.2 ± 14.7	100	<0.001
Total score	87.5 ± 10.2	90	93.9 ± 8.2	98	<0.001

SD: standard deviation

**Table 3:** comparison of PedsQL™ 4.0 generic core scale scores of current study versus published and data

Domain	Normative number mean ± SD	Studied patients and control		
		Group: number	Mean ± SD	
Physical	5962	NS: 100	90.8±13.3	<0.001
	86.9 ± 13.9	Control:100	95.6±10.5	<0.001
Emotional	5961	NS: 100	86.8±13.4	<0.001
	78.2 ± 18.6	Control:100	91.6±14.7	<0.001
Social	5948	NS: 100	95.8±10.9	<0.001
	84.0 ± 17.4	Control:100	97.3±7.9	<0.001
School	5908	NS: 100	74.4±26.4	<0.001
	79.9 ± 16.9	Control:100	90.2±14.7	<0.001
Total	5972	NS: 100	87.5±10.2	<0.001
	82.9 ± 13.2	Control:100	93.9±8.2	<0.001

SD: standard deviation; NS: nephrotic syndrome

**Table 4:** differences between the phenotypes of nephrotic syndrome and the social, school and total score

Parameters	Types of nephrotic syndrome					P value
	Infrequently relapsing (n=23)	Frequently relapsing (n=22)	Steroid dependent (n=17)	Steroid resistant (n=13)	First episode (n=25)	
	Median (interquartile range)					
Physical score	96.8 (84.4-100)	93.7 (89.8-93.7)	96.8 (90.6-100)	100 (90.6-100)	93.7 (87.5-96.8)	0.254
Emotional score	90 (80-100)	92.5 (76.2-92.5)	95 (85-100)	95 (80-100)	85 (70-92.5)	0.183
Social score	100 (95-100)	100 (100-100)	100 (100-100)	100 (100-100)	100 (87.5-100)	0.006
School score	95 (85-95)	87.5 (71.2-87.5)	75 (60-85)	90 (65-97.5)	60 (47.5-87.5)	0.011
Total score	93.5 (82.6-97.8)	90.2 (83.4-95.1)	91.3 (86.9-92.9)	91.3 (89.1-96.7)	82.6 (73.9-91.3)	0.027

**Further analysis:** univariate analysis of variables associated with lower QOL scores revealed that the gender of the patients did not affect scores of all domains (p> 0.05 for all) while the age of the patients at the time of study displayed a negative significant association with the emotional score (r = - 0.04, p=0.01) and positive significant correlation to school functioning (r= 0.192, p=0.006) and duration of illness presented positive significant correlation to the summary score (r = 0.208, p=0.043). No significant variances were reported for summary and domains´ scores among patients who received or did not receive cyclosporine, cyclophosphamide or mycophenolate mofetil (MMF) (p> 0.05 for all).

## Discussion

In children with chronic disorders such as NS, it is increasingly recognized that the burden on HRQOL is not only related to the disease activity and complications but also related to the psychosocial impact of the disease as well as medications´ effects [6,13]. PedsQL™ 4.0 GCS used in the current study is applied for the first time to evaluate QOL in Sudanese children, however, it has been repeatedly used earlier in diverse countries to appraise QOL in different long-lasting illnesses such as diabetes millets [14] and systemic lupus erythematosus (SLE) [15].

In the current study, a male prevalence was stated with SRNS being the least frequent phenotype in the included cohort which is consistent with reports from diverse African nations [16]. Sudanese children with NS reported a high QOL total score, but it is lower than healthy children. Only social domain scores of the patients were not significantly different from controls which are in contradiction to Ruth *et al*. who stated (included only steroid-responsive cases) that only social domain was the only impaired field in patients matched to healthy subjects [6] and a report of Egyptian children with INS (not included newly diagnosed cases) which unveiled all domains of PedsQL™ 4 to be spoiled in patients in comparison to controls [8] and a report from Bangladesh which concluded the social and physical domains to be the most compromised [17]. Ranawaka *et al*. lately stated total score and all domains´ scores to be lower in NS children than in controls but this difference did not grasp statistical significance excluding physical domain [18]. The school functioning of the patients was the lowest which is coherent with the outcomes of Selewski *et al*. [5] and a recent study of 231 Chinese children with NS [19] and a recent report from India [20]. All school children with NS in this study missed school to go to the hospital or because not feeling good however, most of them did not show difficulties in class and did not have trouble with homework.

The physical functioning of our studied group was considerably lower than controls. Li *et al*; reported that most of the children in their report faced a negative impact of NS on physical activity [19]. In a former study of children with SLE, Moorthy *et al*. [15,19] indicated that steroids and other used drugs affected the QOL, leading to. embarrassment of growth, which in turn caused the inability to contribute to physical activities. In the present work gender of the patients did not affect scores of all domains and summary scores which is consistent with findings of the Egyptian cohort of 100 NS children from Egypt [8] but in contradiction with Li *et al*. who described superior scores in males matched to females in all domains [19]. Disparities in sample size, disease period, clinical presentation, and health care services delivered in diverse countries might justify the different consequences between studies.

Regarding the effect of disease phenotype on QOL scores, patients with newly diagnosed NS displayed significantly lower total, social, and school functioning scores compared to other phenotypes. This is inconsistent with a report from Egypt which stated that the SRNS group had the worst scores compared to other phenotypes. However, this study did not involve newly detected patients and included subjects with a longer duration of sickness compared to the existing work [8]. Likewise, a report from Iraq reported IFRNS, and newly diagnosed cases to have healthier QOL in comparison to other clinical types [21]. A recent study from Iran reported no correlation of disease clinical phenotypes and HRQOL scores with only disease duration and number of relapses reported to have negative impact on life quality scores [22].

There is currently no disease specific HRQOL survey for children with NS. PedsQL™-4 GCS has been used on NS children from diverse populations [5,17,23,24], however, its trustworthiness to assess QOL in NS children was lately evaluated in a systematic review involving 10 studies which concluded that the uniformity of the assumptions across the assessed studies accentuates the reliability of non-specific QOL instruments in valuing HRQOL in those children [25]. Recently a trial to create an NS-specific QOL tool was published in 2019, assessed by 11 experts, and applied to 100 NS children [26], however, this tool is not published or authorized yet.

In Africa HRQOL in NS children was evaluated only in Egypt [8] and Nigeria [23,24]. All 3 studies used PedsQL™ 4.0 GCS NS and reported SRNS, long duration of illness and impaired kidney function to be accompanied by poorer QOL scores. Only a few African countries stated data about NS. The steroid-sensitive disease is farther common than steroid-resistant disease although the prevalence of SRNS is higher than reported globally [27]. Hereditary differences along with difficulties in treatment accessibilities add more burden to NS children and their families from low-income African countries which impressively impact their life. This is consistent with Didsbury *et al*. carried out a review article to assess the possible relationship between socio-economic variables and QOL scores of children with chronic diseases and found out a significant relationship [28].

**Limitations of the study:** limitations of the current study are that it is a single-center study and the necessity for follow-up evaluation of variations in scores over a lengthier duration.

## Conclusion

This is the first study to assess QOL in Sudanese children with NS. Sudanese children with NS had lower HRQOL scores compared to healthy children with school functioning being the most spoiled. HRQOL management should be implemented in routine care of children with NS with joined efforts from all health care providers and social employees required. PedsQL™ 4.0 scale is simple and can be used in regular clinical practice to assess QOL in children with NS.

### What is known about this topic

- Idiopathic nephrotic syndrome (INS) in children can impact all features of the life of the children and their families however, no data regarding life quality grades are available from utmost developing countries including Sudan.

### What this study adds

- Sudanese children with NS have lower QOL scores than matching healthy children;
- QOL assessment and management should be part of the daily routine care of Sudanese children with INS.
